# The effect of cigarette smoking history on autonomic and cerebral oxygenation responses to an acute exercise bout in smokers

**DOI:** 10.14814/phy2.14596

**Published:** 2020-10-11

**Authors:** Tegan E. Hartmann, Frank E. Marino, Rob Duffield

**Affiliations:** ^1^ School of Exercise Science, Sport & Health Charles Sturt University Bathurst NSW Australia; ^2^ School of Sport, Exercise and Rehabilitation Faculty of Health University of Technology Sydney (UTS) Sydney NSW Australia

**Keywords:** cycling, nonsmokers, smokers, tobacco

## Abstract

The extent of smoking history is causally linked to adverse cerebro‐ and cardiovascular health outcomes, while conversely, exercise decreases this risk and associated mortality. However, the acute cerebro‐ and cardiovascular responses to exercise in smokers are unknown, and may provide insight to understand chronic adaptation. This study examined the acute heart rate (HR) variability (R‐R intervals) and cerebral oxygenation responses to exercise in smokers compared to nonsmokers. Fifty‐four males classified as smokers (*n* = 27) or nonsmokers (*n* = 27) were allocated into either younger (YSM, YNS) or middle‐aged groups (MSM, MNS). Participants completed 40 min of stationary cycle ergometry at 50% of VO_2peak._ Cerebral oxygenation (near‐infrared spectroscopy) and autonomic function (HR variability) were collected before, during, and after exercise at 0, 30 min, 1, and 4 hr postexercise. The nonsmoker cohort (MNS and YNS) demonstrated higher values for the standard deviation (*SD*) of the R‐R interval (SDNN) and the root mean squared of the *SD* at 1 and 4 hr postexercise versus smokers (*p* < .05). The low frequency (LF) band in YSM was lower than in YNS at 1 hr (*p* < .05). However, LF and high frequency were higher for MNS compared to MSM at 1 hr (*p* < .05). Oxygenated hemoglobin during and following exercise were elevated in NS with values for MSM lower than YSM (*p* < .05). The findings show smoking history can affect cerebral oxygenation during and following an acute exercise bout. Further, following exercise, smokers may exhibit a delay or inhibition in parasympathetic activity.

## Graphical abstract

The findings suggest an effect of smoking history on cerebral oxygenation during and following an acute exercise bout of exercise with nonsmokers demonstrating higher oxygenated haemoglobin compared to that of both young and middle‐aged smokers.

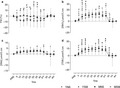

## 1 INTRODUCTION

Tobacco smoking can adversely affect the health of both the cerebro‐ and cardiovascular systems (Ambrose & Barua, 2004; Domino et al., 2004; Terborg et al., 2002). Specifically, acute responses include increased blood pressure, peripheral vascular resistance, heart rate (HR), and altered cerebral and peripheral hemodynamics (Grassi et al., 1994; Siafaka et al., 2007; Terborg et al., 2002). Long‐term smoking is associated with increased vascular permeability and reduced endothelium‐dependent vasodilatation (Bonetti et al., 2002; Domagala‐Kulawik, 2008). However, the development of these states and the effect of the extent of smoking duration (history) have not been reported in any detail. Accordingly, an understanding of the altered cerebro‐ and cardiovascular function that occurs with shorter and longer smoking history may provide insight to explain the changes from chronic tobacco smoking.

Compared to their nonsmoking counterparts, habitual smokers exhibit a heightened risk for cerebro‐ and cardiovascular diseases (Ambrose & Barua, 2004; Yanbaeva et al., 2007). In part, this elevated disease risk may arise from autonomic imbalance as reflected by sympathetic stimulation and increased myocardial contractility (Pasupathi et al., 2009). While chronic tobacco smoke exposure augments sympathetic activity and inhibits vagal tone (Dinas et al., 2013), acute tobacco smoke produces notable increases in sympathetic activity (Karakaya et al., 2007; Kastelein et al., 2016; Mendonca et al., 2011), which may be attributed to the elevated presence of catecholamines in response to nicotine exposure. While chronic smoking is also thought to alter cerebral blood flow, the effects of smoking on cerebral oxygenation are inconsistent. For example, oxygenated hemoglobin is reported to increase (Pucci et al., 2009), while some researchers have reported an increase or no change in deoxygenated hemoglobin (Terborg et al., 2002). These contrasting cerebral oxygenation responses may relate to the complex nature of the chemical composition of tobacco smoke, most notably the presence of carbon monoxide, and noted higher affinity to hemoglobin (Hoyt, 2013; Kastelein et al., 2016; Siafaka et al., 2007).

As opposed to tobacco smoking, exercise has been reported to be effective in the modulation of the ANS and vascular function (Melanson & Freedson, 2001; Pober et al., 2004). Regular exercise may improve cardiac autonomic control via increases in variability, while an acute bout of exercise may elicit a shift in autonomic function toward elevated parasympathetic activity (Pober et al., 2004). Favorable changes in cerebral hemodynamics have been observed with regular exercise training (Ainslie et al., 2008; Murrell et al., 2013), and an acute bout of sub‐maximal exercise induces increases in oxygenated hemoglobin (Ide et al., 1999; Rupp et al., 2013). While the acute exercise responses may contribute to protection of the cerebro‐ and cardiovascular systems, such responses in smokers are seldom reported. Further, it is unknown whether physically active smokers are in turn protected against the adverse cerebrovascular effects of tobacco smoke.

Although many studies have examined the effect of tobacco smoking on physiological function (Ambrose & Barua, 2004; Yanbaeva et al., 2007), very few have examined the sympathetic and microcirculatory outcomes of an acute bout of exercise in a smoker population, let alone the influence of smoking history. Therefore, the purpose of this study was to explore the acute autonomic and cerebral oxygenation responses to exercise in smokers compared to nonsmokers. A secondary aim was to examine the effect of smoking history on autonomic function and cerebral oxygenation outcomes to exercise.

## 2 METHODS

### 2.1 Participants

The study cohort comprised 54 recreationally active males who were either smokers (SM; *n* = 27) or nonsmokers (never; NS; *n* = 27) and were subsequently categorized as either young (YSM, YNS) or middle‐aged groups (MSM, MNS) based on smoking status. All participants reported as apparently healthy and free from any known metabolic, cardiovascular or pulmonary disease, or immunological irregularities and no participants were taking any potentially confounding medications. Participants were matched for age and aerobic fitness as determined by peak oxygen consumption (VO_2peak_). Prior to the commencement of the study, all participants were required to provide written and verbal consent following an outline of all procedures and measures. This study conformed to the Declaration of Helsinki and was approved by the Research in Human Ethics Committee at Charles Sturt University (2012/198).

### 2.2 Baseline testing

Participants reported to the laboratory between 05:00 and 08:00 hr, rested and fasted, including a 10–12 hr abstinence from cigarette smoking, for a baseline testing session. Prior to testing procedures, participants completed the adult pre‐exercise screening system (APSS), a health history and smoking questionnaire, and the Fagerstrom Test for Nicotine Dependence (Heatherton et al., 1991). Anthropometric variables such as stature (Stadiometer: Custom CSU, Bathurst, Australia), body mass (HW 150 K, A & D, Bradford, MA, USA), and waist and hip circumferences (steel tape, EC P3 metric graduation, Australia) were collected based on standardized techniques. Also, a supine dual‐energy X‐ray absorptiometry scan was conducted for the analysis of body composition (XR800; Norland, Cooper Surgical Company, Trumbull, CT, USA). Resting blood pressure was obtained by the asculatory method using an aneroid sphygmomanometer and stethoscope (Welch Allyn, Arden, North Carolina, USA; Perloff et al., 1993) with participants also fitted with a HR monitor (Rs800cx, Vantage NV, Polar, Finland) to measure resting HR.

Participants then completed a graded exercise test (GXT) on an electronically braked cycle ergometer (LODE Excalibur Sport, LODE BV, Groningen, The Netherlands) for the assessment of peak oxygen consumption (VO_2peak_). The younger population began the incremental GXT at 100 W and increased by 25 W every minute until volitional exhaustion, whereas, the middle‐aged population began the GXT at 25 W and increased 25 W every minute. HR was obtained every minute and a session rating of perceived exertion (RPE; modified Borg CR10 scale) was collected at the completion of the GXT.

### 2.3 Exercise protocol

Following the baseline session and 7 d rest period, participants reported to the laboratory in a fasted (including abstinence from smoking) and rested state for the completion of the exercise protocol, which consisted of 40 min of stationary cycle ergometry (Monark 828E, Monark Exercise AB, Varburg, Sweden) at 50% of VO_2peak_. The exercise intensity was calculated as 50% of the pedaling resistance (W) achieved during the GXT. It was converted into kilopond units and set as a fixed intensity for the exercise protocol. Telemetry‐based HR (Rs800cx, Vantage NV, Polar, Finland) and RPE (Borg CR10 scale) were recorded every 5 min during the exercise protocol.

### 2.4 Near‐infrared spectroscopy (NIRS)

Microcirculatory changes in oxygenated ([HbO_2_]), deoxygenated ([HHb]) and total cerebral hemoglobin ([THb]) concentrations were determined by a continuous wave NIRS instrument (Artinis Medical System, Oxymon MKIII, Zetten, The Netherlands). NIRS data were recorded at 10 Hz for the duration of the exercise protocol; a further 3 min recording was obtained at 30 min, 1 hr and 4 hr postexercise. During all NIRS sampling, participants were required to be seated in an upright position and following a stabilization period of 5 min, normalized breathing patterns were ensured. NIRS data collected during the protocol (pre‐, during and postexercise) were normalized against approximately 120 s of baseline data collected before each measurement in a rested state, while seated in an upright position. At data collection, the NIRS probe was placed over the left prefrontal cortex between Fp1 and F3 (international EEG 10–20 system) and placement was adjusted approximately < 5 mm for individual variance. The NIRS probe was affixed with double‐sided adhesives and the inter‐optode distance was fixed at 3.5cm via a black plastic spacer. A modified Beer‐Lambert law was applied to determine oxygenated and deoxygenated heme concentration, based on the absorption coefficient of continuous wavelength infrared light (856 and 794nm) and age‐dependent differential path‐length factors. Total hemoglobin was calculated via the sum of oxygenated and deoxygenated hemoglobin concentrations to indicate regional blood volume. Further, the tissue saturation index (TSI) was calculated as a ratio of oxygenated to total hemoglobin concentrations.

### 2.5 Heart rate variability

Participants were fitted with a HR monitor (Rs800cx, Vantage NV, Polar, Finland) to measure HR and heart rate variability (HRV) during the testing protocol. The collection of HRV was aligned with the collection and timing of NIRS variables, a 3‐min sample was collected at pre‐ and postexercise time points. Following recording, HR files were downloaded to Polar software (Polar Protrainer 5, Polar Electro Oy, Professorintie 5, 90440 Kempele, Finland) via infra‐red; after visual inspection, occasional ectopic beats were identified and replaced with interpolated (linear) adjacent R‐R interval values. HRV analysis was performed using HRV software (Kubios 2.1, Biosignal Analysis and Medical Imaging Group, Finland). Both time and frequency domain analyses were performed. Data from the standard deviation (*SD*) of R‐R interval were interpolated to give values for SDNN, with mean R‐R intervals, and the root mean square of R‐R interval differences (rMSSD) analyzed. A power spectral analysis using Welch's periodgram provided frequency domain parameters (Kubios 2.1, Biosignal Analysis and Medical Imaging Group, Finland). Components of the power spectrum were computed with the following bandwidths: high frequency (HF) (0.15–0.4 Hz) and low frequency (LF) (0.04–0.15 Hz), thus providing the LF/HF ratio.

### 2.6 Statistical analysis

All data are reported as mean ± *SD*. Normal distribution of the HRV data was determined using a Shapiro‐Wilk test and nonnormally distributed data (all HRV variables) were log‐transformed before statistical analysis. Main effects with a Bonferroni correction for condition × time were used to determine the within‐ and between‐group interactions. Where a significant group interaction was observed, simple main effects test with Tukey's post hoc were applied to locate the source of significance. Significance was set at *p* < .05. All statistical procedures were performed using Predictive Analytics Software (PASW) (Statistical Package for the Social Sciences for Windows version 18.0, Chicago, IL, USA).

## 3 RESULTS

**FIGURE 1:**
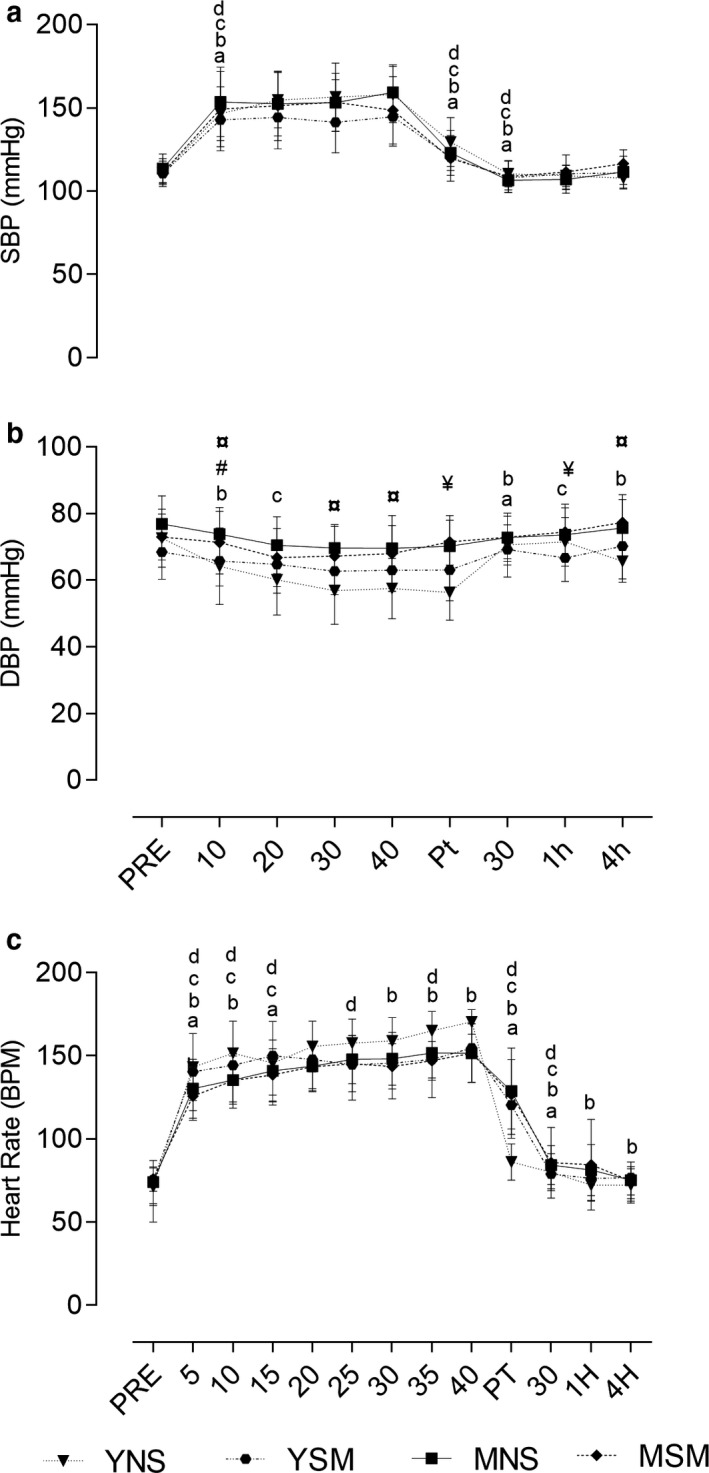
Mean ± *SD* for HR, SBP, and DBP pre, post, 30 min, 1, and 4 hr postexercise for younger and middle‐aged smokers and nonsmokers. Within changes for YSM, YNS, MSM, and MNS are represented by a, b, c, and d, respectively (*p* < .05)

Baseline clinical characteristics for smokers and nonsmokers are presented in Table 1. In terms of smoking history, the MSM group had a longer smoking history (years) (*p* < .001), which was also reflected by pack‐years (*p* = .001). There were no significant differences in the Fagerstrom Test for Nicotine Dependence (FTND) between MSM and YSM regarding the level of nicotine dependence (*p* = .50). There was a main effect for time for within‐group changes across all groups (*p* < .001–.03). Exercise‐induced changes in heart rate and blood pressure can be found in Figure 1.

**TABLE 1:** Mean baseline descriptive, anthropometric, fitness, and smoking characteristics within the smoking (*n* = 27) and nonsmoking (*n* = 27) populations

Descriptive and anthropometric data	YSM	YNS	MSM	MNS
Age (years)	22 ± 1.6	22 ± 1.6	33 ± 7.8^a^	36 ± 6.6^b^
Height (cm)	182 ± 0.07	182 ± 0.06	177 ± 0.07	178 ± 0.06
Weight (kg)	81.78 ± 12.07	86.8 8 ± 16.5	81.22 ± 12.87	90.83 ± 14.51
VO_2_ peak (ml kg^−1 ^min^−1^)	36.67 ± 2.94	39.27 ± 6.06	33.93 ± 8.42	31.62 ± 5.96
Waist to hip ratio	0.86 ± 0.05	0.85 ± 0.10	0.86 ± 0.06	0.91 ± 0.07
Systolic blood pressure (SBP mmHg)	113 ± 9	106 ± 28	116 ± 10	116 ± 9
Diastolic blood pressure (DBP mmHg)	69 ± 9	69 ± 9	76 ± 8	77 ± 8
Heart rate (bpm)	69 ± 11	75 ± 11.	65 ± 12.5	66 ± 8.3
% Fat mass	15 ± 5.8	17 ± 8.1	24 ± 6.8^a^	26 ± 5^b^
Lean mass (kg)	63 ± 9.1	65 ± 12.1	59 ± 6.6	61 ± 5.9
Fat mass (kg)	12 ± 5.3	18 ± 10.2	20 ± 6.8^a^	24 ± 7
Smoking characteristics				
Smoking history (years)	5.21 ± 1.71		14.62 ± 6.29^a^	
Pack‐years	2.86 ± 1.72		12.15 ± 9.08^a^	
Cigarettes per day	12.31 ± 6.54		15.79 ± 7.33	
Fagerstrom test for nicotine dependence Score	2.31 ± 1.32		2.64 ± 1.28	

^a^ Denotes statistically different to YSM (*p* < .05).

^b^ Denotes statistically different to YNS (*p* < .05).

**FIGURE 2:**
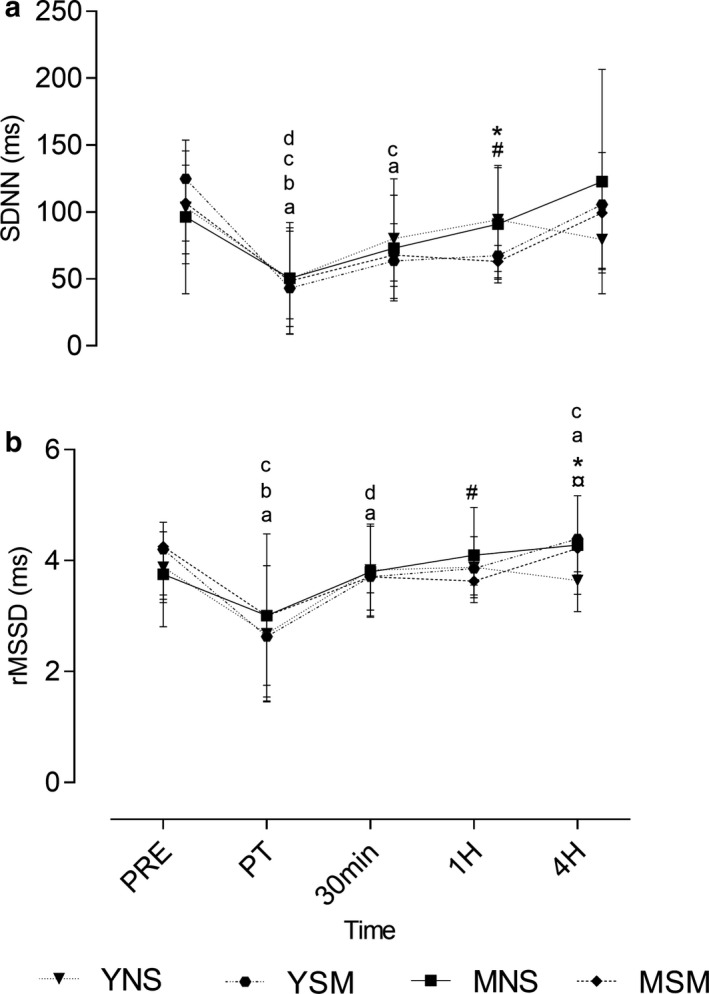
Mean ± *SD* time domain parameters of HRV pre, post, 30 min, 1, and 4 hr post ‐exercise for younger and middle‐aged smokers and nonsmokers. ^*^Represents the significant difference between YSM and YNS (*p* < .05), ^#^represents the significant difference between MSM and MNS (*p* < .05), ¥ represents the significant difference between YSM and MSM (*p* < .05). ¤ represents the significant difference between YNS and MNS (*p* < .05). Within changes for YSM, YNS, MSM, and MNS are represented by a, b, c, and d, respectively (*p* < .05)

The time domain parameters for HRV are presented in Figure 2. Significant main (*F* = 20.24; *F* = 15.50) and interaction (*F* = 1.733; *F* = 1.958) effects were observed for SDNN and RMSSD. The YNS displayed significantly greater SDNN at 1 hr compared to YSM (*p* = .01), while YSM demonstrated higher rMSSD at 4 hr (*p* = .002). For the middle‐aged cohort, there were no differences in time domain parameters (*p* > .05). In comparing smoker groups, rMSSD was higher among YSM at 1 hr (*p* = .05), whereas change in RMSSD for nonsmokers was observed at 4 hr with elevated values in MNS (*p* = .03) compared to YNS. Both MSM and YSM observed within‐group decreases following the exercise protocol in SDNN, followed by an increase to 30 min (*p* = 002). Similarly, rMSSD decreased postexercise and increased thereafter (post‐30 min and 1–4 hr in YSM; 1–4 hr in MSM) (*p* = .009; *p* = .002).

**FIGURE 3:**
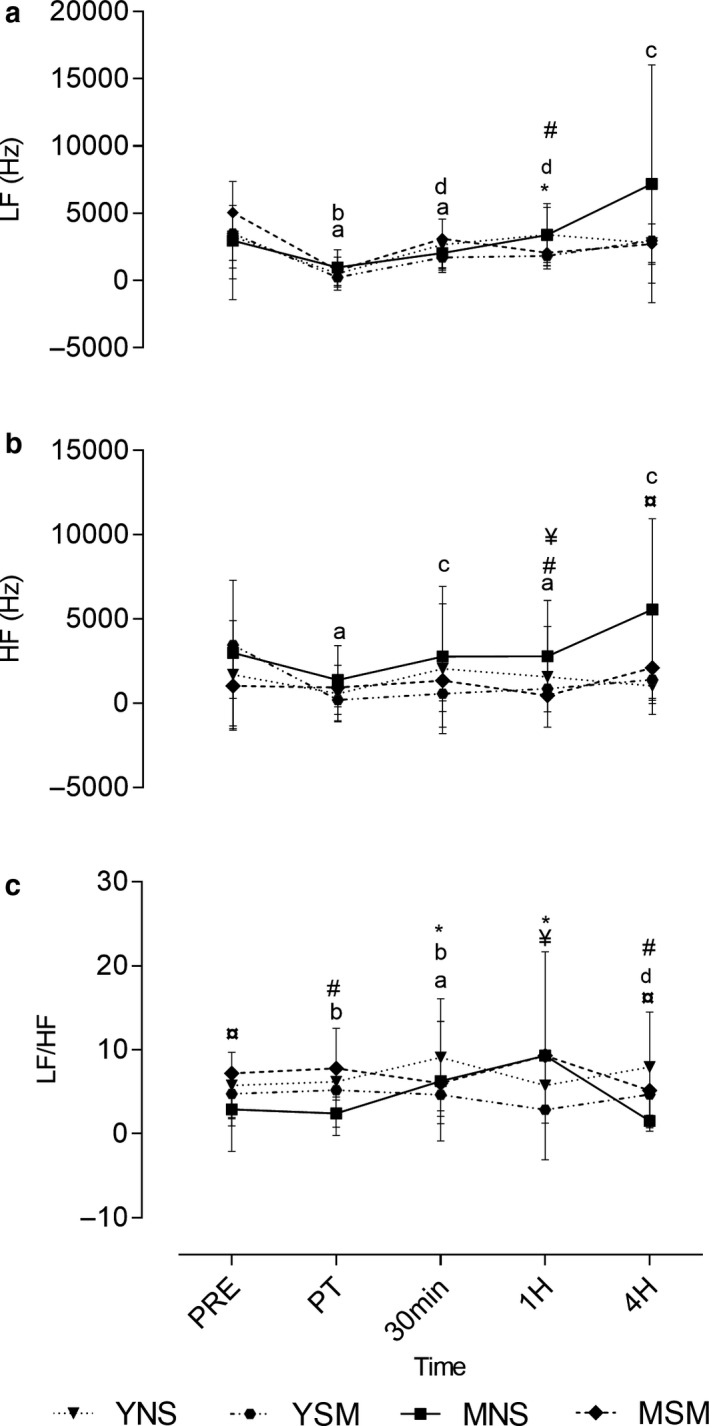
Mean ± *SD* frequency domain parameters HRV pre, post, 30 min, 1, and 4 hr post exercise for younger and middle‐aged smokers and nonsmokers. ^*^Represents the significant difference between YSM and YNS (*p* < .05), ^#^ represents the significant difference between MSM and MNS (*p* < .05), ¥ represents the significant difference between YSM and MSM (*p* < .05). ¤ represents the significant differencebetween YNS and MNS (*p* < .05). Within changes for YSM, YNS, MSM, and MNS are represented by a, b, c, and d, respectively (*p* < .05)

The frequency domain parameters for HRV are presented in Figure 3. Main effects for time were observed for LF and HF (*F* = 29.958; *F* = 20.501), with significant interaction effects for HF (*F* = 2.365). In comparing MSM and MNS, HF was higher among the MNS at pre‐ and postexercise at 30 min, 1 hr, and 4 hr (*p* < .001), whereas the LF/HF ratio at immediate post (*p* = .02) and 4 hr was significantly lower in MSM (*p* = .04). Among YSM, the LF/HF ratio band was lower across all time points compared to YNS (*p* < .001–.006). Between smoker groups, HF was higher in YSM at 1 hr (*p* = .02) and the values for LF/HF were lower in YSM at 1 hr (*p* = .003). For nonsmokers, the HF band at 4 hr was greater among MNS (*p* = .04), whereas the YNS demonstrated significantly greater values for HF and LF/HF at 4 hr postexercise (*p* < .001).

There was a significant within‐group decline in LF postexercise for YSM; values for YSM increased thereafter to 30 min (*p *≤ .001). This was also observed in MSM (*p* = .001–.012); however, for MSM the postexercise elevation in the power of LF continued to increase to 4 hr (*p* = .016). Similarly, LF for YNS decreased immediately postexercise (*p* = .02), whereas for MNS, elevations in LF were observed from postexercise to 1 hr post (*p* < .001–.04). Among young smokers, there was a significant decline in HF postexercise, followed by elevations to 1 hr post (*p* = .006–0.04). YSM and YNS declined in LF/HF 30 min to 1 hr (*p* = .01; *p* = .001–.003), which was observed later for MSM and MNS (1–4 hr) (*p* = .01; *p* = .04).

**FIGURE 4:**
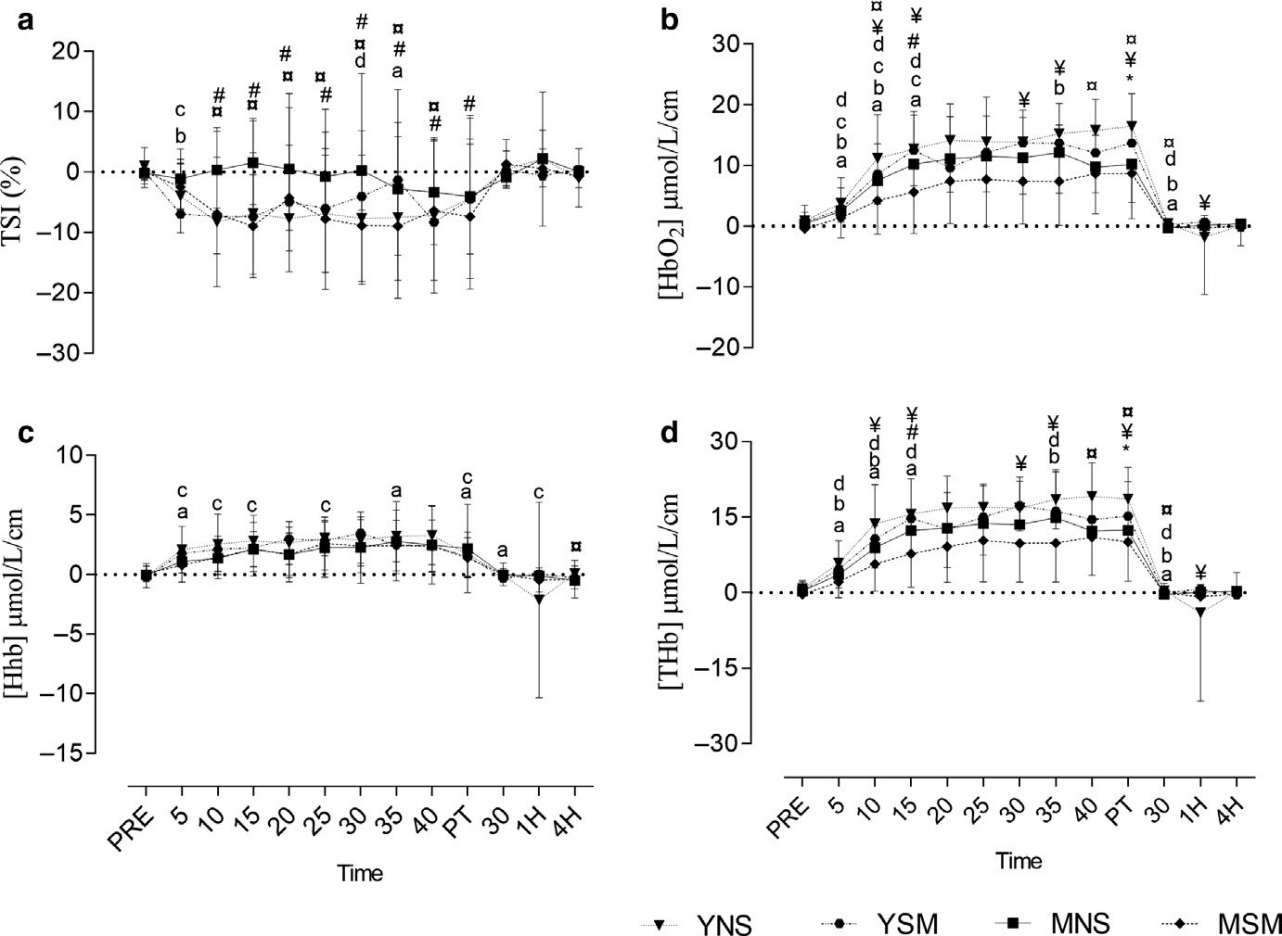
Mean ± *SD* cerebral oxygenation ([HbO_2_]), deoxygenation ([HHb]), tissue saturation index (TSI), and total hemoglobin ([THb]) pre, post, 30 min, 1, and 4 hr postexercise for younger and middle‐aged smokers and nonsmokers. ^*^Represents the significant difference between YSM and YNS (*p* < .05), ^#^ represents the significant difference between MSM and MNS (*p* < .05), ¥ represents the significant difference between YSM and MSM (*p* < .05). ¤ represents the significant difference between YNS and MNS (*p* < .05). Within changes for YSM, YNS, MSM, and MNS are represented by a, b, c, and d, respectively (*p* < .05)

There were no between‐group differences for YSM or YNS regarding TSI or [Hhb] before, during, or postexercise (Figure 4; *p* > .05). However, main effects for [O_2_Hb] and [tHb] were noted (*F* = 33.41; *F* = 32.97) with YNS demonstrating significantly greater concentrations of [O_2_Hb] and [tHb] at 40 min than YSM (*p* = .03). For the middle‐aged cohort, MNS recorded greater TSI from 10, 15, and 30 min during the exercise protocol (*p* = .001–.04). The [O_2_Hb] was greater among MNS at 15 min during exercise protocol (*p* = .03), similarly the [tHb] among MNS was elevated at 15 min (*p* = .03). No between‐group differences for [Hhb] were observed (*p* > .05). In terms of smokers, YSM demonstrated significantly greater [O_2_Hb] and [tHb] during (*p* = .007–.04) and postexercise (post and 1 hr postexercise) (*p* = .03–.05), with no significant differences for TSI or [Hhb] observed pre‐, during, or postexercise (*p* > .05). In comparing the nonsmoker cohort, between‐group significance was observed for TSI (10–30 min during exercise; *p* = .001–.03), whereby YNS demonstrated greater reductions than MNS. The [O_2_Hb] among YNS was greater at 40 to 30 min postexercise than MNS (*p* = .003–.01); [tHb] followed the same trend (*p* = .01–.04). Further, [Hhb] was slightly higher among YNS at 4 hr (*p* = .03). Finally, exercise induced significant within‐group changes in TSI, [O_2_Hb], [tHb], and [Hhb] among all groups (Figure 3).

## 4 DISCUSSION

Our findings suggest that following exercise, smokers may exhibit a delay or inhibition in parasympathetic activity as evidenced by the frequency and time domain parameters of HRV, indicative of altered functioning of the ANS and elevated cerebrovascular disease risk among a smoker population. A further novel finding from this study was that nonsmokers exhibit greater cerebral oxygenation than smokers during and following a bout of low‐intensity exercise. Given that oxygenated hemoglobin and total hemoglobin concentration were significantly lower among MSM, these data suggest an effect of smoking history on [O_2_Hb] during and following an acute exercise bout.

Following a bout of sub‐maximal exercise, cerebral oxygenation is reported to increase (Ide et al., 1999), and may be a mechanism whereby exercise induces favorable effects on cerebral blood flow. Findings from the present study suggest both YNS and MNS demonstrated greater [O_2_Hb] than age‐matched smoking counterparts. Moreover, when comparing the effects of smoking history, the younger group (YSM) had greater [O_2_Hb] and [tHb] both during and after exercise, suggesting an effect of smoking status and smoking history. Similarly, Rupp et al. (2013) found [O_2_Hb] and [tHb] in the prefrontal cortex increased during an exercise protocol that involved 3 × 80 min bouts of cycling at 45% of maximal power output among nonsmokers. While the effects of smoking status and duration have not been extensively researched in relation to [O_2_Hb], previous research suggests that acute cigarette smoke exposure (single cigarette, 0.9 mg nicotine) has been shown to increase [O_2_Hb] and [tHb] during and following cigarette consumption (Terborg et al., 2002). As we have previously reported (Kastelein et al., 2016), acute tobacco smoking increases TSI and [HHb] during cigarette consumption, followed by declines postcigarette consumption for [HHb] in MSM but not YSM. These findings indicate an effect of smoking history on cerebral microcirculatory responses to acute smoking. The current study revealed habitual smoking status might result in lower [O_2_Hb] in response to an acute bout of exercise, and that longer smoking history may exacerbate this response. Such exercise‐induced responses may result from decreased vascular reactivity and exercise‐induced vasodilation (Wüst et al., 2008), as chronic smoking has previously been reported to decrease the bioavailability of nitric oxide (Heitzer et al., 1996). Thus lower [O_2_Hb] due to smoking and length of smoking history may indicate a precipitating mechanism preceding the development of cerebrovascular diseases.

Autonomic imbalance, as characterized by reduced vagal activity, is associated with increased mortality (Thayer et al., 2009). Differential autonomic responses are inferred from inter‐beat variation for time domain parameters, whereas the frequency domain indicates the change in length of R‐R intervals (Achten & Jeukendrup, 2003). Particular lifestyle factors, such as tobacco smoking and physical activity, are considered strong modulators of this autonomic balance (Thayer & Lane, 2007). The findings from the present study indicate that all groups exhibit parasympathetic inhibition immediately postexercise, followed by a return to baseline values. However, the magnitude of the response was greatest among the nonsmoker group, with elevated parasympathetic activity at 1 hr as represented by the time domain parameters, SDNN and RMSSD. These responses suggest that chronic smoking or smoke exposure causes a delay in vagal tone postexercise, and thus prolonged sympathetic activity. In support, the frequency domain parameter of the HF band, which reflects parasympathetic activity, was higher in MNS at pre‐exercise and from 30 min postexercise to 4 hr postexercise. The adverse outcomes of smoking on autonomic function have been previously reported, as Barutcu et al. (2005) compared HRV parameters in long‐term heavy smokers and nonsmokers, suggesting evidence of blunted vagal modulation in smokers. Similarly, Levin et al. (1992) observed chronic cigarette smoking to be associated with lower HRV, as indicated by the mean R‐R interval. A single bout of smoking is capable of inducing alterations in autonomic balance as we have previously reported an acute bout of smoking to result in vagal withdrawal, indicative of sympathetic hyperactivity (Kastelein et al., 2016). The collection of current evidence suggests that vagal inhibition or delay, particularly evident in the time domain parameters, may be indicative of sympathetic hyperactivity among habitual smokers. However, further investigation is required to substantiate this hypothesis. Ultimately, such findings may be indicative of the adverse changes in autonomic tone as observed with chronic tobacco smoking.

Despite our novel findings, the following limitations need to be acknowledged when interpreting these results. First, it should be noted that the exercise intensity of 50% VO_2peak_ would be considered a relatively low intensity for an acute exercise bout. However, given the duration of exercise (40 min), sedentary and smoking nature of the population, this intensity was selected to ensure appropriate contraindications were controlled for (Kastelein et al., 2016). Second, recent research outlines limitations regarding the use of the RS800CX heart rate monitor for the assessment of HRV at rest and during exercise (Tsitoglou et al., 2018). Accordingly, our results should be interpreted with an awareness of the variability of HRV parameters from this technology.

In conclusion, the present study suggests nonsmokers have greater cerebral oxygenation in response to acute low‐intensity exercise. Among the smoker cohort, an effect of smoking history may be evident, with MSM demonstrating lower oxygenated and total hemoglobin than their younger smoking counterparts. Moreover, while all groups exhibited vagal withdrawal as a response to exercise, smokers present with an inhibition or delay of vagal tone following an acute bout of exercise, which may be suggestive of sympathetic hyperactivity. When considering previous research on the effects of smoking on cerebral blood flow and autonomic balance (Domino et al., 2004; Dinas et al., 2013; Terborg et al., 2002), the current findings may highlight factors that precipitate increased disease risk in cigarette smokers. Further, such findings may be used by clinical exercise practitioners to inform exercise prescription for smokers of both a shorter and longer smoking history.

## 5 DATA STATEMENT

The data that support these findings are available upon reasonable request from the corresponding author.

## CONFLICT OF INTEREST

The authors declare no conflict of interest.

## AUTHOR CONTRIBUTIONS

TH, FM, and RD were involved in the conceptual design of the study. TH collected and analyzed the data and drafted the manuscript. FM and RD provided critical feedback on the manuscript.

## ETHICAL STATEMENT

This study conformed to the Declaration of Helsinki and was approved by the Research in Human Ethics Committee at Charles Sturt University (2012/198).
